# Selected Advancements in the Management of Atrial Fibrillation from the Year 2021

**DOI:** 10.19102/icrm.2022.130107

**Published:** 2022-01-15

**Authors:** James A. Reiffel

**Affiliations:** ^1^Columbia University, New York, NY, USA

**Keywords:** Anti-arrhythmic drugs, atrial fibrillation, pharmaceuticals, rhythm management

Most often, highlights in a pharmacologic section of a journal regarding the year that just passed will focus on the development or approval of new therapeutic agents, new uses for older agents, or new efficacy or safety information. However, given the absence of approval of any new anti-arrhythmic drugs (AADs) or oral anticoagulants (OACs) in 2021, for this commentary, I have instead chosen to discuss the following several specific thematic advancements in the management of atrial fibrillation (AF) this past year.

1. The downgrading of sotalol for AF in the 2020 European Society of Cardiology (ESC) AF guidelines
2. More appreciation regarding the “pill-in-the-pocket” (PITP) therapy for pharmacologic cardioversion of recent-onset AF
3. An important international survey of rhythm management approaches to AF in both Europe and the United States (US) and by cardiologists versus electrophysiologists
4. An underappreciation that ablation versus AADs for AF requires consideration of endpoints beyond AF recurrences

## The downgrading of sotalol for atrial fibrillation in the 2020 European Society of Cardiology atrial fibrillation guidelines

There are four main pillars of consideration regarding the therapy of AF.^1,2^ First is the evaluation and treatment of underlying and contributory comorbidities and conditions—including hypertension, heart failure, ischemia, sleep apnea, obesity, autonomic imbalance, and more. Some have termed this “upstream therapy.” Reducing these and their effects on the left atrium (LA) can and has reduced the degree of atrial myopathy that frequently underlies the initiation and progression of AF and its thromboembolic and hemodynamic consequences.^1,2^ Second is the reduction of the typically rapid ventricular response to AF such that the ventricular rates are more akin to what the rates would be at the same level of activity in sinus rhythm.^1,2^ Doing so will alleviate or reduce some of the symptoms commonly present in association with AF as well as prevent the development of a tachycardia-induced ventricular cardiomyopathy. Third is the administration of OACs to patients with AF and elevated stroke risk markers, so as to reduce the likelihood of stroke and systemic embolism.^1,2^ Fourth is rhythm control—that is, therapy directed at the restoration of sinus rhythm and the minimization of AF recurrences/overall AF burden.^1,2^ Rhythm control is pursued to reduce AF symptoms that persist despite rate control as well as to prevent or slow AF progression. Importantly, as AF, once rate-controlled and appropriately anticoagulated, should inherently have a very low residual mortality risk, algorithms to guide rhythm-control therapy advanced by both the American College of Cardiology/American Heart Association/Heart Rhythm Society (ACC/AHA/HRS) and the ESC have focused upon the principle of safety first, rather than efficacy first.^1,2^ More specifically, the 2020 ESC guidelines state: “The aim of AAD therapy is to improve AF-related symptoms. Hence, the decision to initiate long-term AAD therapy needs to balance symptom burden, possible adverse drug reactions, and patient preferences … safety should dictate both the initiation and continuation of AADs.”^2^ Similarly, with respect to the most effective yet most toxic AAD for AF, the ACC/AHA/HRS guidelines state: “Owing to its potential toxicities, amiodarone should only be used after consideration of risks and when other agents have failed or are contraindicated.” Accordingly, the safest AADs should be tried first, when not contraindicated, and, for most patients, ablation has followed at least one trial of a more benign AAD option when reasonable.^1^

The safety considerations regarding AADs have focused mainly upon organ toxicity and ventricular pro-arrhythmic risks, although drug–drug interactions, negative inotropic effects, and potential for depression of sinus nodal and conduction system function also require consideration.^1,2^ Of the AADs currently guideline-recommended for AF, it is organ toxicity (along with numerous drug interactions) that has relegated amiodarone to its last-line position. In contrast, dofetilide, dronedarone, flecainide, propafenone, and sotalol have minimal if any substantial organ toxicity concerns. With respect to ventricular pro-arrhythmia, the two class Ic agents, flecainide and propafenone, have almost no risk in the structurally normal (or near-normal) ventricle but can produce sustained ventricular tachycardia/fibrillation in structurally abnormal ventricles, such as those with scar, fibrosis, and ischemia. Accordingly, they are indicated as first-line options when significant ventricular disease is absent but are contraindicated when significant ventricular disease is present. In contrast, dronedarone has been remarkably safe in its clinical trials with respect to ventricular pro-arrhythmia in patients both without and with structural heart disease (SHD) except in those with severe or recently decompensated heart failure—where increased mortality has been present, particularly if co-administered with digitalis. Accordingly, dronedarone appears in the algorithms as a first-line option in patients with and without heart disease, except for the setting of significant or unstable heart failure. As for dofetilide and sotalol, these two agents prolong the QT interval and can produce torsades de pointes (TdP) polymorphic ventricular tachycardia with potential for hemodynamic collapse and degeneration to ventricular fibrillation.^1,2^ While this risk is greater in the presence of substantial ventricular hypertrophy, it is also present in normal hearts. In the American guidelines, they are listed as first-line options in patients without SHD.^1^ However, because of their TdP risk, I believe they should be listed as second-line therapies following (alphabetically) dronedarone, flecainide, and/or propafenone, which appear to carry less risk. In the American guidelines, dofetilide, dronedarone, and sotalol are also listed as first-line drugs in patients with SHD (whereas flecainide and propafenone are now absent), except that sotalol should be avoided when significant systolic heart failure is present, and both dofetilide and sotalol should be avoided in the presence of left ventricular (LV) hypertrophy or more than mild renal dysfunction. Here again, when dronedarone is available as an option, I believe it is a safer choice to use it prior to sotalol or dofetilide. In Europe, dofetilide has never been approved for use and thus is not in the ESC guidelines. Importantly, as of the 2020 ESC guidelines, sotalol has been downgraded to a second-line option because of its pro-arrhythmic risk,^2^ which is consistent with my previous comments. The exact wording is: “Sotalol may be considered for long-term rhythm control in patients with normal LV function or with ischaemic heart disease if close monitoring of QT interval, serum potassium levels, CrCl, and other pro-arrhythmia risk factors is provided. (Class IIb, level of evidence A).”

## More appreciation regarding “pill-in-the-pocket” therapy for pharmacologic cardioversion of recent-onset atrial fibrillation

By definition, when AF is not permanent, it is intermittent; and, when intermittent, it may take the form of persistent episodes requiring cardioversion for termination or paroxysmal episodes (paroxysmal AF [PAF]) which, by definition, are self-terminating (typically within 24–48 hours and by definition within seven days). At times, because of the severity of the associated symptoms, PAF is also cardioverted prior to allowing time (eg, days) for self-termination. In patients with symptomatic AF episodes, as per the prior discussion, preventing, shortening, or reducing the number of recurrences is pursued with rhythm-control strategies that encompass AADs, ablation, or both. However, for patients with relatively infrequent episodes of AF that are tolerable for at least several hours and have no associated ischemic or hemodynamically significant impairment, the use of an AAD only at the time an episode begins would seem preferable to a daily AAD regimen or an invasive ablative procedure. This strategy of acute AAD administration to produce rapid termination of a new AF episode has been termed “PITP”.^3^ Conversion rates with PITP therapy in clinical trials have generally been in the 70% to 80% range within four to eight hours, which shortens the episodes significantly versus spontaneous conversion times. At what frequency of AF to use PITP therapy versus daily AAD therapy or ablation should be determined by the patient, with considerations involving symptoms, quality of life, lifestyle, travel, economics, and other patient preference factors.

Many physicians I have encountered have little to no experience with this therapeutic method, although it is recognized in both the American and European AF guidelines. The 2014 AHA/ACC/HRS guidelines state: “Flecainide, dofetilide, propafenone, and intravenous ibutilide are useful for pharmacological cardioversion of AF or atrial flutter provided contraindications to the selected drug are absent. Administration of oral amiodarone is a reasonable option for pharmacological cardioversion of AF. Propafenone or flecainide (PITP) in addition to a β-blocker or non-dihydropyridine calcium channel antagonist is reasonable to terminate AF outside the hospital once this treatment has been observed to be safe in a monitored setting for selected patients.”^1^ The atrioventricular (AV) nodal blocker is added so as to prevent 1:1 AV conduction if AF changes to atrial flutter prior to reverting to sinus rhythm, which can occur with class IA and IC AADs. Nonetheless, neither of these two drugs should be used if the patient has any contraindication to the use of a class IC AAD. Similarly, the 2020 ESC guidelines note: “In selected outpatients with rare PAF episodes, a self-administered oral dose of flecainide or propafenone is slightly less effective than in-hospital pharmacological cardioversion but may be preferred (permitting an earlier conversion), provided that the drug safety and efficacy has previously been established in the hospital setting. An atrioventricular node-blocking drug should be instituted in patients treated with class IC AADs (especially flecainide) to avoid transformation to AFL with 1:1 conduction.”^2^

The reason that propafenone and flecainide are listed for PITP therapy whereas dofetilide and amiodarone are not is because of the time frame of their action. For the symptomatic patient, rapid, rather than slow, termination is preferable. That being said, a PITP regimen should ideally be relatively quick to achieve its effect and have a short washout time in case of any adverse events. Therefore, the PITP method uses AADs with rapid uptake kinetics, short times to tissue penetration, short half-lives, and taken orally. Given their kinetics, propafenone and flecainide fulfill these criteria, whereas dofetilide, dronedarone, and amiodarone do not. Please note that sotalol is not listed here, as oral sotalol has a rather low likelihood of terminating an AF episode despite reasonable efficacy for the prevention of recurrent ones. Also effective for PITP therapy, though off-label and tested in fewer patients in clinical trials, is ranolazine.^3^ The advantage of ranolazine is that it has no contraindication for ischemic heart disease (where it is approved for angina therapy) or heart failure. Also, as suggested previously, as PITP regimens primarily target recurrent PAF, the drug should be one that can be administered at home once verified as effective and safe under observation.

The dosing strategies of propafenone, flecainide, and ranolazine for PITP therapy are as follows:

- Flecainide: 300 mg single dose (consider 200 mg if weight is < 70 kg)
- Propafenone: 600 mg single dose immediate-release formulation (consider 450 mg if patient weight is < 70 kg)
- Ranolazine: 2,000 mg (given as a single dose, or two 1,000-mg doses given no more than four hours apart)

The mean conversion times with flecainide and propafenone are just under four hours and that with ranolazine (in my experience) is less than six hours.

I introduced this section with a heading that commented on more appreciation for PITP therapy, but how can this be quantified? In 2021, an online survey of 629 practicing cardiologists and electrophysiologists in the US and Europe who manage AF was performed, titled “Anti-arrhythmic Interventions for Managing Atrial Fibrillation (AIM-AF): A Physician Survey in the United States and Europe.” The survey comprised 86 questions on physician demographics, AF types, and treatment practices. As how the PIPT approach is used in physicians’ practices has never been formally examined, the questions concerning this treatment strategy were numerous. The results of this portion of the survey were presented at the AHA Annual Scientific Sessions in November 2021.^4^ In short, the survey revealed that PITP is used by American and European physicians, respectively, in 24% and 19% of their AF patients. The frequency of PIPT use was also greater in patients with PAF without SHD (41%) than those with SHD (16%) and among electrophysiologists than general cardiologists. For AF without SHD, class IC AADs were used most often (flecainide, 77%; propafenone, 32%), but there was notable inappropriate use of amiodarone (13%) and sotalol (13%), the latter more in the US. For AF with SHD, class IC use appropriately diminished considerably. PIPT was given with a rate-control agent (new or chronically, β-blocker ; calcium channel blocker) in 71%, while 29% gave PIPT AADs without concomitant rate-control agents. Optimal arrhythmia frequencies for PIPT were felt to be: monthly (13%), every two to three months (46%), every four to six months (26%), every seven to twelve months (11%), and yearly or less (4%), with no notable differences between the US and Europe, or cardiologists and electrophysiologists. Thus, given that PITP therapy is not rare, the review herein seemed appropriate to cover. For the reader interested in learning yet more about PITP therapy, including its history, clinical experience in the literature, and more, I would suggest reading the detailed review by Reiffel and Capucci in the American Journal of Cardiology this past year.^3^

## An important international survey of rhythm management approaches to atrial fibrillation in both Europe and the US and by cardiologists versus electrophysiologists

The AIM-AF survey covered many more areas of AF management than just PITP. The results publicly presented/published so far (aside from the PITP data stated previously) include the following.^5–8^

The AHA/ACC/HRS and ESC guidelines are widely accepted references for selecting anti-arrhythmic therapies for patients with AF. One goal of the AIM-AF survey was to assess the extent of guideline use among different AF physicians across Europe and the US. It was also developed to learn if there are discrepancies between clinical practice regarding AAD use and ablation versus guideline recommendations.^5^ There were 629 respondents, with a similar distribution between Europe and the US (51% vs. 49%, respectively) and slightly more cardiologists compared with electrophysiologists (57% vs. 43%, respectively). Respondents had an average of 14 years’ experience in their specialty, with 80% having a subspecialty in AF. Overall, 45% prescribed drug treatments and performed ablation, while 55% prescribed drug treatments but referred for ablation.

Guidelines were reported as the most important non-patient factor influencing AF therapy choice for 53% of respondents. For 28%, they were the second most important non-patient factor. Although, as noted already, guidelines favor safety over efficacy as the pivotal factor in choosing each step in rhythm-control therapy, efficacy was considered as the more important factor by 49% of respondents in contrast to safety by 33% of respondents. In deciding upon the pursuit of rhythm-control therapy, AF type and symptoms were the most important considerations, with heart failure (HF) listed as the third most important reason to select rhythm control over rate control.^5,8^

Deviations from guideline indications were not uncommon.^5,6^ Amiodarone was the most frequently chosen AAD in each of the many heart disease categories queried (59%–81%) except for minimal or no SHD, where it was still chosen in 23%. Class IC AADs were chosen by 6% to 8% of respondents in coronary disease categories, despite being contraindicated in such, as well as by 8% for patients with HF with reduced LV ejection fraction (HFrEF) and 18% for HF patients with preserved LV ejection fraction (HFpEF). Sotalol was chosen by 27% of respondents for patients with reduced HFrEF, by 35% for patients with LV hypertrophy, and by 22% for patients with renal insufficiency (the percentages varying among US and European physicians and cardiologists and electrophysiologists). Outpatient initiation of sotalol was reported by 56% of respondents (and in the US, dofetilide initiation as an outpatient was noted by 17%). Overall, the use of sotalol was consistently higher among US respondents compared with European respondents across a range of comorbidities—perhaps reflective of the recent downgrading of sotalol in the 2020 ESC guidelines algorithm. Also, despite safety concerns, only 50% to 64% of respondents routinely followed renal function and only 51% to 54% routinely followed electrolytes during chronic therapy with sotalol. Most surprisingly, and without apparent indication, AADs were used by 35% and 38% of respondents, and ablation by 8% and 13%, respectively, for asymptomatic and subclinical AF.^5^ We have no solid data to support these latter practices. The authors concluded that the AIM-AF study was the first survey to extensively explore physicians’ treatment decisions with regard to anti-arrhythmic therapies in patients with AF. Deviations from guidelines were common, with treatments frequently used in inappropriate indications or in clinical contexts that have the potential to compromise patient safety. The results of this survey suggest an underappreciation of the pharmacology and safe use of AADs. Importantly, this is not the first paper that has noted important discrepancies between guideline recommendations and clinical practice patterns.^9,10^ While certainly there are individual patient situations or characteristics that can lead to such discrepancies, overall, it would appear that educational opportunities to improve patient care are clearly present.

When considering more specific AF presentations,^8^ oral AADs were preferred over ablation for infrequent, mildly symptomatic PAF (58% of respondents), while ablation was preferred over AADs for frequent, symptomatic PAF (62% of respondents). Thirty-nine percent of respondents preferred ablation over AADs as a first-line therapy in patients with AF plus HFrEF (as is consistent with the 2021 Scientific Statement from the AHA regarding the management of AF in patients with HFrEF),^11^ whereas 20% preferred AADs first line in the same patient group. In both patients with HFrEF and HFpEF, amiodarone was used less in the US than in Europe (71% vs. 91%), whereas sotalol was used more in the US than in Europe (25% vs. 17%). Ablation use is higher in patients with recurrent AF as compared to first-episode AF, ie, 15% with first episode, 61% with recurrent AF, and 71% with recurrent AF that failed multiple AADs.^7^

## An underappreciation that ablation versus anti-arrhythmic drugs for atrial fibrillation requires consideration of endpoints beyond atrial fibrillation recurrences

For the last decade and a half, a growing number of trials have been performed that have compared catheter ablation (CA) versus AAD therapy as first-line treatment for AF—predominantly for PAF in non-elderly patients. In these trials, there has been growing consistency supporting CA as the superior approach for reducing recurrent AF. These have included the following trials (virtually all just including PAF, the last two of which were published in 2020 and 2021):

1. Radiofrequency Ablation vs Anti-arrhythmic Drugs for Atrial Fibrillation Treatment (RAAFT) in 2005^12^ performed in 70 patients with a primary efficacy endpoint of any symptomatic or asymptomatic AF episode of 15 seconds or more on three 24-hour Holter recordings and monthly telephone loop recorders during 12 months’ follow-up; the mean age was 53 years, the mean LA size was 4.14 mm, and no CHADS_2_ or CHA_2_DS_2_-VASc score was reported.
2. Medical Anti-arrhythmic Treatment or Radiofrequency Ablation in Paroxysmal Atrial Fibrillation (MANTRA-PAF) in 2012^13^ performed in 294 patients with a primary efficacy endpoint of AF burden, expressed as the percentage of time in AF on each and all seven-day Holter recordings during 24 months’ follow-up; the mean age was 55.5 years, the mean LA size was 4.0 mm, and the CHADS_2_ score was zero to one point(s) in 89%.
3. RAAFT-2 in 2014^14^ performed in 127 patients with a primary efficacy endpoint of time to first recurrence of symptomatic or asymptomatic AF, atrial flutter, or atrial tachycardia of at least 30 seconds on scheduled electrocardiograms (ECGs) and trans-telephonic monitors during 24 months’ follow-up; the mean age was 55 years, the mean LA size was 4.15 mm, and the mean CHADS_2_ score was 0.6 points.
4. Early Aggressive Invasive Intervention for Atrial Fibrillation (EARLY-AF) in 2020^15^ performed in 303 patients with a primary efficacy endpoint of any atrial tachyarrhythmia of at least 30 seconds and/or repeat ablation on scheduled ECGs and an implanted loop recorder with daily transmissions during 12 months of follow-up; the mean age was 58 years, the mean LA size was 38 mm, and the mean CHA_2_DS_2_-VASc score was 1.9 points.
5. Sustained Treatment of Paroxysmal Atrial Fibrillation (STOP-AF) in 2020^16^ performed in 203 patients with a primary efficacy endpoint of any atrial tachyarrhythmia of at least 30 seconds, initial procedure failure or need for repeat ablation, AF surgery, cardioversion, and/or use of AADs after the blanking period during follow-up of 12 months; the mean age was 60.5 years, the mean LA size was 38 mm, and the CHA_2_DS_2_-VASc score was zero to one point(s) in 45%.
6. Cryoballoon Ablation vs. Anti-arrhythmic Drugs: First-line Therapy for Patients with Paroxysmal Atrial Fibrillation (Cry-FIRST) in 2021^17^ performed in 218 patients with a primary efficacy endpoint of any atrial tachyarrhythmia of more than 30 seconds on scheduled ECGs and Holter monitors during 12 months of follow-up; the mean age was 61 years, the mean LA size was 38 mm, and the CHA_2_DS_2_-VASc score was zero to one point(s) in 45%.

In each of these relatively small trials, CA was better than AADs at reducing recurrent atrial tachyarrhythmias. In meta-analyses of these trials,^18,19^ ablation was also associated with less use of health care resources. Please note, however, the relatively young ages, low embolic risk scores, and small LA sizes in these trials, and determine how your patients in your practices compare with these trial subjects. Notably, in these trials, ablation was not associated with lower mortality or fewer strokes/systemic emboli. Also not provided in these trials are data on how patients in the CA groups were managed after they reached their first efficacy endpoint; that is, how many underwent additional ablation or how many had AADs added to their post-CA management either during the remainder of the trial (only one to two years of total follow-up in these trials) or after the trial ended. This is important as AF is a chronic disorder, and for many, the ultimate management is a combination of ablation coupled with AADs rather than an either/or choice. Fortunately, some additional information regarding these issues is available from the Catheter Ablation vs. Anti-arrhythmic Drug Therapy in Atrial Fibrillation (CABANA) trial.^20^

The CABANA trial (published in 2019 but discussed here for contrast) was a much larger and longer trial (the median duration of follow-up was 48.5 months) than any of the above. While it did compare AF recurrences in patients treated with CA versus those treated with a drug regimen (both AADs and rate-control-only options were allowed in the drug arm), the primary efficacy outcome was a composite of death, disabling stroke, serious bleeding, or cardiac arrest. A total of 2,204 symptomatic patients with AF aged 65 years and older or younger than 65 years with one or more risk factors for stroke were enrolled. The median age was 68 years; 37.2% were women; 43% had PAF and 57% had persistent AF; the median CHA_2_DS_2_-VASc score was three points, with only 18% scoring zero to one point(s), though only 155 had a history of HF; 89.3% completed the trial. Patients had to be eligible to receive at least three drug options, but 81% had been tried previously on at least one AAD, suggesting the possibility of a somewhat drug-resistant population. Of the 1,108 patients randomized to CA, 1,006 received CA while 102 did not (patient or physician refusal or insurance issues); 215 received repeat ablation(s). AADs were taken by 45% of the CA patients during the course of the trial after the blanking period, with 26.5% still on an AAD at their last point of follow-up. Of the 1,096 patients assigned to drug therapy, 1,092 (99.6%) received drug therapy: 853 received rhythm and rate control, 123 received rate control only, 116 received rhythm control only, and seven received no drug treatment. Five hundred and forty-five patients received one AAD, 296 received two, 106 received three, and 22 received four or more different AADs over the course of the trial. Three hundred and one patients (27.5%) ultimately crossed over to receive CA. To determine AF recurrence rates, patients were provided with an ECG event recorder for chronicling symptomatic events; underwent 24-hour autodetect, full-disclosure, real-time recordings on a quarterly basis; and obtained 96-hour Holter recordings every six months regardless of symptoms.

In the intention-to-treat analysis, the primary endpoint occurred in 8.0% of patients in the ablation group versus 9.2% of patients in the drug therapy group (hazard ratio [HR], 0.86; 95% confidence interval [CI], 0.65–1.15; P = .30). Among the secondary endpoints, outcomes in the ablation group versus the drug therapy group, respectively, were 5.2% versus 6.1% for all-cause mortality (HR, 0.85; 95% CI, 0.60–1.21; P = .38), 51.7% versus 58.1% for death or cardiovascular (CV) hospitalization (HR, 0.83; 95% CI, 0.74–0.93; P = .001) (virtually all due to reduced CV hospitalization), and 49.9% versus 69.5% for AF recurrence (HR, 0.52; 95% CI, 0.45–0.60; P < .001).^20^ In the prespecified treatment received analyses (which has to be considered in the context of the biases inherent in this type of analysis), the HR for CA versus drug therapy with respect to the primary endpoint was 0.67 (95% CI, 0.50–0.89; P = .006). For all-cause mortality, the corresponding HR was 0.60 (95% CI, 0.42–0.86; P = .005), and for death or CV hospitalization, the HR was 0.83 (95% CI, 0.74–0.94; P = .002).^20^

Recall, however, that the above superiority of ablation as seen by the treatment-received analysis occurred with a non-negligible percentage of the ablation patients having received an AAD during the post-blanking follow-up period. In the “real world,” this is not unusual. As reported by the AIM-AF survey study,^7^ AADs are used empirically post-ablation for AF prophylaxis by 34% of physicians for 3–6 months. They are also given by 34% for a single recurrence, by 32% for bridging to re-ablation, and by 34% for long-term therapy. Even more use AADs long term post-ablation if the ablation was for persistent AF, with 40% specifically stating amiodarone.^7^

Importantly, CABANA is not the only trial to suggest that CA may improve outcomes such as survival versus AAD therapy. The others are trials that have been performed in HFrEF patients, as are well summarized in a 2021 Scientific Statement from the AHA regarding the management of AF in patients with HFrEF.^11^ This document notes: “In multiple randomized clinical trials in recent years, catheter ablation for atrial fibrillation in patients with HFrEF has shown superiority in improving survival, quality of life, and ventricular function and reducing heart failure hospitalizations compared with anti-arrhythmic drugs and rate-control therapies. This has resulted in a paradigm shift in management toward nonpharmacological rhythm control of atrial fibrillation in HFrEF … Given these data and the fact that only limited therapeutic options are available for patients with advanced HF, it is plausible to consider CA as a first-line therapy for patients with AF and HFrEF, recognizing, however, that patients with severe HFrEF may derive less benefit.”^11^

While this superiority of CA over AADs regarding mortality appears clear in selected patients with HFrEF from the trials reviewed in the 2021 AHA Scientific Statement, we are not yet at the point where we can make such definitive conclusions regarding a preferred approach to rhythm control in other AF populations. Nonetheless, we must begin to appreciate that simply reducing AF recurrence is not the optimum endpoint to be assessing in CA versus AAD trials. While it certainly is one important endpoint—which would be most accurately assessed as AF burden using continuous monitoring methods rather than time to first recurrence using intermittent ECG assessment—we need to learn whether more important adverse outcomes, such as death, embolism, hospitalization, and costs, are reduced by one specific therapeutic approach or another. This is analogous to determining the winner of a baseball game. It is not the number of hits one team gets versus the other (eg, number of AF events), it is the number of runs scored that determine the winner (fewer deaths, strokes, etc.). And, just as in baseball where substitutions are allowed at the discretion of the team manager, substitutions in the form of changing AADs, switching to ablation, adding an AAD after ablation, etc. are all part of the patient care reality. It is not a choice of AADs versus CA, it is when to use which, when to switch to the other, and when to use a combination of the two. Importantly, we should make these decisions data-driven when possible.
